# Preterm twins with antenatal presentation of Pearson syndrome

**DOI:** 10.1515/crpm-2021-0083

**Published:** 2022-02-01

**Authors:** Leonor Castro, Ana C. Ferreira, Álvaro Cohen, Israel J. Macedo, Teresa Tomé

**Affiliations:** Pediatric and Neonatal Intensive Care Unit, Hospital Central do Funchal, Av. Luís de Camões, nº 57 – 9004-514 Funchal, Madeira, Portugal; Metabolic Diseases Unit, Hospital Dona Estefânia, Centro Hospitalar Universitário de Lisboa Central, Lisboa, Portugal; Prenatal Diagnosis Unit, Maternidade Dr. Alfredo da Costa, Centro Hospitalar Universitário de Lisboa Central, Lisboa, Portugal; Neonatal Intensive Care Unit, Maternidade Dr. Alfredo da Costa, Centro Hospitalar Universitário de Lisboa Central, Lisboa, Portugal

**Keywords:** fetal anemia, hyperammonemia, pancytopenia, Pearson syndrome, preterm, twins

## Abstract

**Objectives:**

Pearson syndrome is a mitochondrial cytopathy with multisystemic involvement that typically presents in infancy and has poor prognosis. We aim to present a case that is distinct due to the timing of presentation and associated anomalies.

**Case presentation:**

We report the case of preterm monochorionic twins with transfusion dependent fetal anemia that had post-natal multisystem dysfunction which led to the diagnosis of Pearson syndrome.

**Conclusions:**

This case highlights the possibility of antenatal presentation of Pearson syndrome, which should be considered in cases of severe fetal anemia without an apparent cause.

## Introduction

Pearson syndrome (PS) is a rare multisystem mitochondrial disorder that was first described in 1979 by Pearson et al. It is caused by a large-scale mitochondrial DNA deletion that causes defective oxidative phosphorylation and usually presents within the first year of life [1], [2], [3] with varying multisystem dysfunction, including the classically described refractory sideroblastic anemia and exocrine pancreatic dysfunction [4]. Prognosis is very dismal and up to two thirds of surviving infants can evolve to Kearns-Sayre Syndrome [1, 3, 5].

Fetal presentation of PS is extremely rare – to our knowledge there are only three reported cases [3, 6]. With this report we aim to present the case of monochorionic twins with severe transfusion dependent fetal anemia as initial presentation of PS.

## Case presentation

A 32-year-old primigravida with spontaneous monochorionic diamniotic twin pregnancy, was referred to our center at 24 weeks and two days gestational age due to suspected twin–twin transfusion syndrome. The parents were nonconsanguineous and healthy. The pregnancy was complicated with gestational diabetes, which was adequately controlled with diet. Ultrasound of both twins showed subcutaneous oedema, ventricular hypertrophy, and ascites, with peak systolic velocity on medial cerebral artery (PSV-MCA) above 99th percentile (both umbilical vein and ductus venosus Doppler were normal). The second twin had also ventricular septal defect and hypospadias. Twin–twin transfusion syndrome was not confirmed. The hemoglobin values were 1.7 g/dL on the first fetus (F1) and 2.7 g/dL on the second fetus (F2) and both were transfused on admission, following corticosteroid administration for fetal lung maturation. There was a rise in hemoglobin to 8.2 g/dL and 9.7 g/dL on F1 and F2, respectively. A second transfusion was performed 13 days later (at 26 weeks and one day gestational age). Pre-procedure hemoglobin values were 8.1 g/dL (F1) and 9.5 g/dL (F2), and post-procedure hemoglobin were 15.5 g/dL (F1) and 12.7 g/dL (F2). Follow up ultrasound showed normalized PSV-MCA and oedema. Hematological (isoimmunization and haemoglobinopathies) and cytogenetic (karyotype and Array-CGH) studies were negative. Cytomegalovirus IgM and IgG were positive, yet IgG avidity was high. Other TORCH studies, including Parvovirus B19 were negative. Fetal growth restriction was identified at 28 weeks in both fetuses and an elective caesarean-section was performed at 31 weeks and one day, due to altered ductus venosus Doppler in F1. Both babies were born with extreme pallor, no respiratory movements and bradycardia. Their Apgar index at first, fifth and tenth minutes, was 4, 6, 8 and 1, 8, 9, for F1 and F2 respectively. Birthweights were 1,345 g (F1, 24th percentile) and 1,010 g (F2, 5th percentile). They were both intubated and ventilated and transferred to our NICU.

## Evolution

Baby one (B1) was male and had no external abnormalities. Baby two (B2) was also male and had hypospadias and bilateral cryptorchidism. Echocardiogram revealed biventricular hypertrophy on B1 and ostium secundum atrial septal defect, large perimembranous ventricular septal defect and abnormal right coronary artery arising from anterior descending artery on B2. Despite morphological differences, both twins evolved in a very similar course: they were ventilator-dependent, required repeated courses of vasoactive support and pharmacological closure of patent ductus arteriosus (which was unsuccessful on B2 but clinical stability for surgical procedure was never achieved). They were hypotonic with absent Moro and suction reflexes and brain ultrasound revealed parenchymal microcalcifications, periventricular echogenicity, and grade two bilateral intra-periventricular hemorrhage.

They had severe refractory agenerative microcytic anemia (nadir hemoglobin 6.8 g/dL and 5.9 g/dL, Mean Corpuscular Volume 79.6 fL and 80.6 fL on B1 and B2, respectively) and leukopenia since day one. Severe thrombocytopenia ensued on the first two days. Peripheral blood smears were inconclusive. Direct antiglobulin test was negative. Urine Cytomegalovirus was negative as were other infection screenings. Both babies had refractory coagulopathy (elevated thromboplastin and activated partial thromboplastin times with extremely low fibrinogen), that, along with thrombocytopenia, resulted in digestive, pulmonary, urinary, and central nervous system hemorrhage. Support therapy was provided with multiple erythrocyte, platelet, plasma, and cryoprecipitate transfusions along with phytomenadione and filgrastim. Albumin was low but hepatic enzymes and creatine kinase were normal. Serum amylase levels were undetectable, and both had transitory hyperglycemia.

Renal tubular disfunction was also present, with polyuria, glucosuria, proteinuria, hematuria and severe electrolyte imbalance with hyponatremia, hypocalcemia, and hypophosphatemia, that required multiple corrections. Mild hypokalemia was identified only on B2 and was easily corrected. Abdominal and renal ultrasound were normal.

Both babies had persistent metabolic acidosis with low plasma bicarbonate (nadir 10.0 mmol/L on both), high anion gap and hyperlactatemia (maximal lactate 133 mg/dL and 149 mg/dL on B1 and B2, respectively) since day one, which required multiple bicarbonate corrections and eventually bicarbonate infusion. They also had hyperammonemia (maximum 336 μmol/L and 321 μmol/L on B1 and B2, respectively) and started sodium benzoate and protein restriction. Levothyroxine was prescribed for hypothyroidism.

Newborn blood spot screening test on day five discarded organic aciduria. Pyruvate was normal. Plasma amino acid chromatography revealed elevated alanine, tyrosine, methionine, lysine, proline and hydroxyproline on B1 and raised glutamate on B2. Urine organic acid chromatography showed elevated lactate, pyruvate, and acetate.

The florid clinical picture gave rise to the suspicion of PS. Due to the size and instability of the babies it was impossible do perform a bone marrow aspirate. Genetic study was performed only on B1 due to extremely difficult blood draw on B2. Next-generation sequencing panel didn’t identify any mutation associated with nuclear mitochondrial cytopathies. Mitochondrial DNA study in leukocytes identified a large deletion (5 kb), confirming PS.

The babies deteriorated progressively with hypotension, persistent oral and intestinal bleeding, and anasarca. After discussion with the parents, supportive care was withdrawn on day 27 and 31 for B1 and B2, respectively. Comfort measures were provided. The babies died at days 27 and 34 of life, respectively.

## Discussion

PS is a mitochondrial disease that is caused by single large-scale deletions of mitochondrial DNA [7]. It is usually sporadic, although in 4% of the cases single mitochondrial DNA deletions may be inherited from the mother [8]. Pregnancy and birth are usually uneventful [7] and symptoms typically begin within the first year of life. It is a multisystemic disease, that affects bone marrow, heart, kidneys, liver, pancreas, gastro-intestinal system, central nervous system, endocrine organs, eyes, skin, musculoskeletal system, and others [3, 8]. Malformations are uncommon, although there are reported cases of cleft lip and palate, hypospadias, and polydactyly [7, 8].

Manea et al., in 2009, reviewed 79 cases of PS and found that neonatal onset occurred in 42% of the cases. Six patients had fulminant multiorgan involvement at birth and two of them presented with severe hydrops fetalis. At the end of the follow up period 55% had deceased [3].

Prenatal symptoms of PS have been reported in only three cases: Rötig et al., in 1990, published the first case of a term baby that had severe anemia and hydrops fetalis, neutropenia, thrombocytopenia and mild metabolic acidosis. Parenteral nutrition precipitated fatal liver failure at 25 months of age [4]. Li et al., in 2003, published the second case of a baby with hydrops fetalis diagnosed at 38 weeks gestational age that had severe anemia and moderate thrombocytopenia at birth but evolved favorably with therapeutic support and had hematological recovery at 15 months old [9]. The third case was presented by Gussi et al., in 2009, and reported a case of fetal anemia and growth arrest at 32 weeks gestational age that progressed to myocardial hypertrophy and pericardiac effusion at 36 weeks. The baby had medullar aplasia at birth, but hematologic spontaneous recovery ensued after nine months of supportive transfusion therapy. At 20 months, metabolic acidemia and failure to thrive ensued. There was no further report on evolution [6].

Our case is the first report of PS that presents antenatally in twins with dependent transfusion anemia at a very early gestational age. Prompt intervention by obstetric team prevented evolution to hydrops fetalis however, even with highly intensive supportive therapy, the babies did not survive.

Nevertheless, we experienced some difficulties regarding our diagnostic approach: since the babies received two intrauterine erythrocyte transfusions and multiple post-natal transfusions, red blood cell indices and peripheral blood smear were difficult to interpret. Anemia in PS is usually macrocytic, severe and transfusion dependent [2, 4, 7], but in our case both babies had microcytic agenerative anemia. Ring sideroblasts and vacuolization of bone marrow precursors are classically observed [4, 7], but we were not able to perform a bone marrow study due to the size and instability of the babies. Neutropenia is usually moderate to severe [7] and there is a variable degree of thrombocytopenia [4]. In our case they were both severe.

Renal tubular dysfunction and Fanconi syndrome have both been described in association with PS, as well as hypospadias. Cryptorchidism has not been associated with PS but may be related to prematurity, since 30% of premature male infants are born with uni or bilateral cryptorchidism [10].

Regarding cardiac anomalies, ventricular hypertrophy has been described [7, 8], but not atrial or ventricular septal defects or coronary artery anomalies, as in our case.

Although neurological examination is usually normal in neonatal period, inability to suck and axial hypotonia have been described [7, 11]. Basal ganglia calcifications and diffuse white matter lesions have been reported, among other central and peripheral nervous system anomalies [8]. Our patients had parietal and temporal calcifications, but not on basal ganglia. Periventricular echogenicity was probably related to prematurity. Ophthalmological observation, in pursuit of corneal and retinal changes associated with PS, was not possible due to clinical instability.

Concerning pancreatic exocrine dysfunction, both babies had low levels of amylase, but it is known that newborns have low levels or complete absence of pancreatic amylase [12]. Fecal elastase was not measured because the babies had persistent bloody stools. Diabetes is found in up to 27% of the patients [7] but our patients had only transient hyperglycemia. Hepatic disfunction was evidenced by hypoalbuminemia and altered coagulation, since liver enzymes were normal. Exocrine pancreatic dysfunction and bone marrow alterations were not possible to identify but still we had a strong suspicion and proceeded with the study of mitochondrial DNA.

Hyperlactatemia is typically present (1.3–5 times increased) as is urinary lactate [7]. Urinary pyruvate may also be increased, as well as citric acid cycle intermediates, such as fumarate and malate [7, 13, 14]. We identified increased urinary lactic, acetic, and pyruvic acids, which is in accordance. Typically, alanine is raised in 90% of the cases [7], although that was only identified on B1. Ammonia levels are usually low to normal in PS [2, 13] but the babies had persistent hyperammonemia, perhaps due to an association of prematurity, gastrointestinal bleeding, prolonged parenteral feeding, and ongoing severe disease. Hypothyroidism has been associated with PS [5, 15], but it is also present in 35–85% of very preterm babies [16].

Therapy is supportive and includes blood products, nutritional support (including fat soluble vitamins and pancreatic enzymes), bicarbonate and dichloroacetic acid for lactic acidosis, treatment of endocrinological problems and infections [5, 7]. Granulocyte colony-stimulating factor (filgrastim) has been used to increase neutrophil counts [7]. Bone marrow transplant, which can only correct hematological problems, has been experimented with poor outcomes [7, 13].

The prognosis is dismal, with initial series reporting death of the majority within the first three years of life [17], but most recent publications report death occurring between the ages 5 to 11 years [7], possibly due to better therapeutic support and understanding of the disease. Children usually succumb due to intractable metabolic acidosis, renal or hepatic failure, hemorrhage, and sepsis [3, 7, 17].

Considering the difficulties in treating this disease and its prognosis, it is important to provide families with appropriate genetic advice. Genetic counselling is extremely difficult and should be particularly cautious [4, 7], since this is for the vast majority a sporadic disease but in a small percentage may be maternally inherited (maternal germline mosaicism) [5]. Moreover, there may be considerable variation in the mutated DNA load and the outcome for each pregnancy is difficult to predict. Preimplantation genetic testing [5] and antenatal diagnosis via amniocentesis and chorionic villus sampling could be used in mitochondrial DNA disorders [18] but prenatal test results cannot reliably predict phenotype [5].

### Take home messages

With this case we intend to highlight the possibility of antenatal presentation of PS, which was previously reported in very few cases. It is possible that such early presentation, with the need for antenatal transfusions, may predict more severe post-natal course and worse outcome. Additionally, hyperammonemia, atrial and ventricular septal defects and coronary artery anomalies have not yet been reported in PS. There is a vast (and expanding) spectrum of manifestations in PS, but the association of pancytopenia, hyperlactatemia and multi-organ dysfunction should prompt the investigation of this disease.
